# Designing fair annual bonus formulations for workers: A case study of the state-owned enterprise cement holding in Indonesia

**DOI:** 10.1057/s41599-022-01471-3

**Published:** 2022-12-10

**Authors:** Effnu Subiyanto, Roy Kurniawan

**Affiliations:** Department of Postgraduate School, Faculty of Magister Management, Widya Mandala Surabaya Catholic University (UKWM), 60265 Surabaya, Indonesia

**Keywords:** Business and management, Business and management

## Abstract

A bonus is an additional annual incentive for labour, as part of remuneration package systems, that goes hand-in-hand with a *tantième* for boards of commissioners and directors. This practical business is common in industrial relations for maintaining a spirit of productivity and loyalty. However, practices to distribute bonuses remain undisclosed and unknown, according to the views of labour. Without an earlier mutual agreement between parties, consisting of labour representatives and management, management unilaterally decides a specific bonus formulation that takes effect immediately. The management ignored suggestions and advice from labour as its partner; worse, there were no discussions between parties in advance. There are no equal labour and management rights to build a better industrial relations climate. We employed mixed methods and conducted multidisciplinary studies to determine cluster and bonus pool allocation with relevant peers to investigate how to build a fair bonus formulation. We adopted a target-based method rather than an outcome-based framework after several exercises. The most important finding is that the bonus formulation, configured from the annual bonus, utilised three underpinning percentiles (P): P-25, P-50, and P-75 implying the lowest, medium, and highest total remuneration for labour, respectively. We determine that success indicators in developing fair bonus formulation stand on how high total remuneration has been achieved by labour towards the median level of peers. The higher the value passed over the median line, the higher the success rate. The present findings contribute to building fair annual bonus formulations in the cement sector; however, other industrial sectors can use them with adequate adjustments.

## Introduction

In addition to normative wages and salaries, labour (workers in this study) have a right to claim annual bonuses as part of the fair compensation system. To date, a few studies have supported a general understanding of annual bonuses as part of a rewards and compensation system that is initially considered appropriate for management only (Alkebsee et al., 2022). In this case, the management is the board of commissioners (BOCs) and board of directors (BODs). In Indonesia, this additional incentive to management is known as *tantième*, which was granted because of meeting the annual performance stipulated by contract management between BODs and BOCs with the Ministry of State-owned Enterprises (SOEs). In contract management, the *tantième* formulation has thus far not been publicly disclosed due to confidentiality. The clauses stated in contract management are somewhat different in terms of interpretation and understanding, distinctive between one SOE to others. In short, only those who reached BODs or BOCs could feel the actual substance of their contract management.

The challenge is how to develop a fair annual bonus formulation between parties. Bonus formulation for management is precisely decided by contract management; therefore, this study will not discuss contract management. Instead, this study only builds bonus formulations for workers. The consideration is that the workers have rights to be appropriately fulfilled because their efforts to accomplish targets have been made. Even though this obligation is clearly written in the 2-year work contract between workers and management, management generally reconducts several exercises to convince themselves that the additional disbursement will not ruin corporate performance (Liao and Han, 2019; Makowski-Komura and Bebenroth, 2020; Sim et al., 2021). Unfortunately, with a small amount of specific interest, workers will frequently be disappointed. Thus, fair formulation and transparency have become increasingly important in this matter.

The case of state-owned enterprises grouped within cement holdings in Indonesia is rather specific, especially the Semen Indonesia Group (SIG) case as an object of this study, which is the first corporation to develop this issue. As a holding, the SIG employs thousands of workers. Some are located in the headquarters in Jakarta, and many others are stationed in several subsidiaries’ offices throughout Indonesia. The workers in headquarters only handle management issues, such as regulating policies or exercising strategies. In contrast, significant workers stationed in subsidiaries (well-known as operating companies or opcos) are concentrated on serving daily operations and productions. The whole group of SIG combines 36 subsidiaries, five of which are cement makers, and the other 31 business units are noncement makers. The total number of embedded workers in the SIG was ~9359 people (H1-2022). In this context, the SIG, representing large corporations, addresses the relationship between the two types of management, which are organising costs and bargaining costs (Lunnan et al., 2019; Grund and Hofmann, 2019).

This study is made from open and long public discussions by proposing several practical business models to develop a better annual bonus formulation. This issue is routine as the main subject of conflict between workers and management every year and is costly in terms of time and industrial relations (Sánchez et al., 2020; Baadel et al., 2020). The workers have frequently accused management of applying nongovernance industrial relations practices because the allocation bonus pool was unilaterally decided without previous consent as a mutual agreement between the parties. Unfair allocation of the bonus pool would certainly disappoint workers when, despite the effort and hard work made to achieve the annual target, the amount received was much less than expected at the end of the year.

## Justification

Annual bonuses are a distinctive matter for Indonesian workers. A bonus is given as an appreciation because the yearly performance has met the target. Moreover, a bonus is the same language of equal rights, while BODs and BOCs may have claimed their *tantième*, and workers can do so to claim their bonuses. The big questions are how to deliver bonuses fairly, transparently, and in compliance with the rules of governance. This study is the first work to investigate and explore mechanisms to provide annual bonuses as fairly as possible. Amid the limited literature, percentile methods have been developed as industrial guidance in treating this issue commensurately. This study explores, reviews, and analyses annual reports to determine how the value of the bonuses delivered to workers in cement industries in Indonesia. These results were clustered into three types of percentiles (P) in P-25, P-50, and P-75. The higher percentile indicates higher bonuses disbursed to their workers, which meant that the industries were stable and mature. The medium percentile determined that the industries were in the developing stages in which annual bonuses were given at the middle level. The lowest percentile indicated lower bonuses for workers, generally for industries categorised as new entrants. From the actual empirical work done by peers, the position of each cement industry could be determined, where they stand, and the value of bonuses to be appropriately distributed. This method is a way to resolve routine conflicts and disputes between management and workers that initially came from unfairness, unilateralism, and misunderstanding.

## Research gap

At all times, determining the fairness of the bonuses allocated to workers remains a big question. Limited studies are the source of these problems; consequently, industrial disputes and conflicts remain without a solution. These are fundamental gaps that must be carefully comprehended. To date, the valuable findings of previous studies have been unable to help resolve these conflicts. There is no definition yet of what industries should do to address these issues correctly and adequately. The research results have not reached a new finding as an offer to build a better solution for resolving disputes due to claiming ‘unfair allocation’ in the bonus distribution. This study provides best practices from empirical business experiences to fill the research gap and bring mutual benefits to workers, societies, and industries.

## Theoretical background

Management organises operations to control businesses. Their departments formulated, tested, and implemented policies and strategies as part of corporate decision-making (Christie and Dubrowski, 2021). An important factor is a mechanism as the tool of management controls (Rikhardsson et al., 2021). Sageder et al. (2019) addressed the complex management controls for managing the group of holdings consisting of headquarters and subsidiaries. According to Osma et al. (2022), some management controls are linked to earnings management. Bonuses as part of earnings are within the scope of management control. The challenge is how to treat this subject fairly and effectively. Should the bonus be given on a cash or noncash basis? Cash is clear, while noncash, such as building a better working environment to increase job satisfaction, is another form of bonus (Rojikinnor et al., 2022). Other forms of bonuses are compensation, rewards, or incentives.

Assessing quality objectives is also a part of management control (Lau et al., 2021). The tasks should be measurable, objective, and fair, as the results will impact the remuneration systems (Bugdol and Jedynak, 2022). The principle is that workers should receive equal pay for equal work performed (Kosheleva and Aguilar, 2022; Škrinjarić, 2022). Advanced workers are paid appropriately and fairly according to the formula agreed upon.

An annual bonus is a distinctive strategy originally used to treat the highest level of executives (Harris and Brown, 2021; Al-Faryan, 2021; Schmid and Baldermann, 2021; Graziano and Rondi, 2021; Alkebsee et al., 2022; Chen and Hassan, 2022; Pan et al., 2022; Liew et al., 2022; Sandberg and Andersson, 2022). In two-tier management systems, the levels mentioned above should address the BOCs and BODs. The US introduced the arrangement of CEO-to-worker pay ratios in advance, but unexpected results occurred due to an increase in the wage gap (Schulz and Flickinger, 2020; Johnson, 2022).

Bonuses are the different subjects that emerged to compensate for the impact of income gaps. A bonus is an additional income, a nonlinear payment system, a variable, or nonfixed, with a penalty or consequence (Cai et al., 2022). This case can be awarded because of specific considerations as an additional condition, one of which was successfully meeting the annual corporate target. The other prerequisites for claiming bonuses are performance indicators that surpass an agreed target (Keßels, 2021; Liao and Han, 2019). Thus, bonus arrangements are limited by strict conditions that might be incidentally added, such as if performance is possible and management is willing to accommodate the issue (Al Hadabi et al., 2019).

Generally, workers in developing countries are not automatically awarded decent annual bonuses (Andalib et al., 2019). In Indonesia, one of these welfare indicators of workers must be met to raise this concern to comply with stipulated laws (Andalib et al., 2019; Subiyanto, 2020b; Subiyanto, 2021). However, the legal outline is just higher than the poverty line; therefore, the obligations are limited for addressing below the line of poverty (Maleka et al., 2021; Kojanic, 2021; Wulansari, 2021). In this case, workers are uneasy about claiming their rights legally. It is not easy to fight for better welfare.

Some countries pay bonuses as part of their additional annual earnings. Although European countries have experienced a decrease in total revenues due to the great recession, arguments still support bonuses due to the changes in workers and job characteristics (Sławińska, 2021). If developed countries experienced a decrease in workers’ total earnings, striving for an unclear definition in developing countries would be a lengthy effort for workers with an uncertain outcome.

However, it must be carefully considered that understanding bonuses as part of adjustment compensation is important. This understanding helps build a better climate in industrial relations (Latta, 2019; Alpar, 2020; Beck et al., 2020; Brück et al., 2021). Corporate governance and culture have improved, including tightening the relationship between management and workers (Assenso-Okofo et al., 2021). The income gap will be lessened due to better governance (Baixauli-Soler et al., 2021).

Therefore, building a suitable bonus formulation has become an important topic. The substantive argument is that workers expect their total income to reach at least the median level of their peers. These arguments are based on principles of equality and fairness based on performance (Ziano et al., 2022). Financial indicators are suggested to determine formulas to include variables such as earnings after tax (EAT), revenue, and operating expenses (Kong et al., 2022) at the headquarters. Other necessary variable operations, such as total volume produced or complete services given, are used for measuring the performance at the subsidiaries’ offices or opcos. Other studies have been determined based on standard practices. The variables also generally suggest net profit (EAT), revenue, production volume, and operating expenses (Ababneh et al., 2019; Rini and Subiyanto, 2021).

## Methodology

In this study, we develop a qualitative methodology to address these issues. We employed mixed methods and conducted multidisciplinary studies. Descriptive analyses were then built to explain the findings. This study was conducted in 2021, as all 27 cement makers in Indonesia had published their annual reports. This issue was not officially known before 2021, but it received attention in 2021 due to the industrial conflicts and disputes registered in the Industrial Settlements Courts in Jakarta in 2021.

First, we explored a population of 27 cement makers to determine their clusters. The clusters were divided into three classifications: new entrants, developing, and mature industries. Simultaneously, other data and information were exercised by analysing their annual reports. Although some of the cement makers were still private companies, each has established official business websites containing quarterly and annual reports, from where we obtained the data and information.

As a result of step one, we built Table 1 to determine the clusterisation. The cluster was calculated based on annual reports divided into three categories, each based on an annual base, total cash, and total remuneration. The categories were built using the following equations:

**Table 1:** Clusterisation of cement makers in Indonesia.

No.	Corporate names	Brands	Installed capacities	Cluster
1	PT Solusi Bangun Andalas, Aceh	Semen Andalas	3 million tpa	1
2	PT Semen Padang	Semen Padang	5 million tpa	3
3	PT Semen Baturaja	Semen Baturaja	1.8 million tpa	4
4	PT Solusi Bangun Indonesia, Narogong	Dynamix	3 million tpa	1
5	PT Indocement Tunggal Perkasa, Citeureup	Tiga Roda	9 million tpa	1
6	PT Semen Bima (STAR)	Semen Bima	3 million tpa	4
7	PT Solusi Bangun Indonesia, Cilacap	Dynamix	3 million tpa	1
8	PT Semen Merah Putih, Banten	Semen Merah Putih	1.8 million tpa	4
9	PT Semen Garuda (Juishin)	Semen Garuda	1.8 million tpa	4
10	PT Semen Jakarta, Ciwandan	Semen Jakarta	1.8 million tpa	4
11	PT Semen Puger Jaya Raya Sentosa, Jember	Semen Puger	1.8 million tpa	5
12	PT CONCH Kalimantan Selatan	Conch	5 million tpa	4
13	PT Indocement Tunggal Perkasa, Tarjun, Kalsel	Tiga Roda	3 million tpa	2
14	PT Semeru Surya Semen	Semen Surya	1.8 million tpa	4
15	PT Semen Indonesia (Persero) Tbk., Gresik	Semen Gresik	3 million tpa	2
16	PT Semen Gresik, Rembang	Semen Gresik	1.8 million tpa	3
17	PT Semen Kupang, NTT	Semen Kupang	0.2 million tpa	5
18	PT Semen Tonasa, Pangkep, Sulawesi Selatan	Semen Tomasa	5 million tpa	3
19	PT Semen Bosowa, Maros, Sulawesi Selatan	Semen Bosowa	3 million tpa	3
20	PT Semen Jawa (Siam Cement Group)	Semen SCG	1.8 million tpa	3
21	PT Solusi Bangun Indonesia Tbk, Tuban	Dynamix	3.6 million tpa	2
22	PT Haohan Cement Indonesia, Serang, Banten	Semen Serang	0.12 million tpa	4
23	PT Conch North Sulawesi Cement, Bolaang Mongondow, Sulawesi Utara	Conch	3 million tpa	4
24	PT SDIC Papua Cement Indonesia, Maruni, Manokwari, Papua	Conch	1.8 million tpa	5
25	PT Sunfook Cement Indonesia (Hippo Cement), Bojonegoro, Serang, Banten	Semen Hippo	3 million tpa	4
26	PT Conch Barru Cement Indonesia, Sulawesi Selatan	Conch	3 million tpa	4
27	PT Semen Kaltim	Semen Kalimantan	1.8 million tpa	5

*tpa* ton per annual.

(1) Annual base = (12 months*salary base) + religious holiday allowance + leave allowance + official allowance + location allowance;

(2) Total cash = annual base + bonuses; and

(3) Total remuneration = total cash + other benefits (if available).

From Table 1, we built a clusterisation based on total annual income. We made a specific notation that the lower cluster meant higher benefits, and cluster-1 is the highest total remuneration delivered. We made several tables to show the current portrayed peer system based on clusterisation in Indonesia. Figures that explain categories can be seen in Table 2.

**Table 2:** Scale workers’ benefit cement corporations (Rp million).

Description	Cluster-1	Cluster-2	Cluster-3	Cluster-4	Cluster-5
Annual income	700–1300	450–850	300–450	150–250	125–200
Total cash	1100–2000	600–1200	300–650	250–310	175–250
Total remuneration	1200–2100	700–1300	400–700	250–400	250–300

To simplify interpreting data and information, we presented Table 3 as a resume and built peer methods consecutively in cluster-1, cluster-2, cluster-3, cluster-4, and cluster-5.

**Table 3:** Peer clusterisation cement corporations in Indonesia.

Description	Cluster-1	Cluster-2	Cluster-3	Cluster-4	Cluster-5
Number of corporations	4	3	5	11	4

Second, we employed mathematical equations to develop models and build charts to help show where cement makers stand. Third, we made a novel model and a description and analysed why the model fits to solve open issues. We propose a novel development and better formulation for annual bonuses by combining these two methods.

**Fig. 1:**
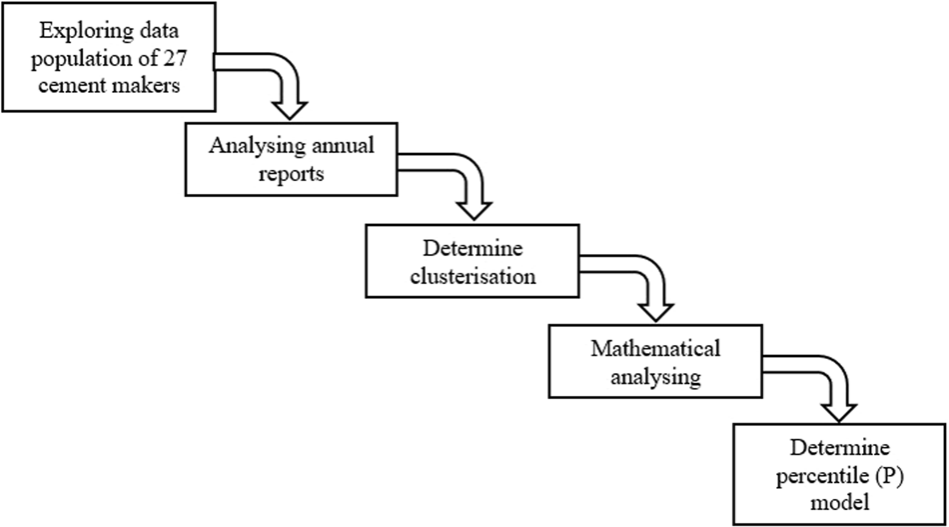
The mechanism developed in this study. Indonesia has total of 27 cement corporations in 2022. We did a systemic flow of methods which at first exploring all populations, analysing annual reports, determining cluster, mathematical exercising, and finally defining the percentile (P) model.

In total, we built the mechanism of this study through five stages. The first was the exploration of the population of 27 cement makers, simultaneously accessing annual reports as the second stage, determining clusterisation as the third stage, exercising mathematical analysis to build a better annual bonus formulation as the fourth stage, and finally choosing the percentile (*P*) model as the fifth stage. Figure 1 illustrates the mechanism used in this study.

This study established two types of bonus formulations that should be designed to address issues at the headquarters and opcos. Because the number of opcos reached 36 entities, the formulation should be typical and identical considering the target they accomplished and the operating expenses they consumed. This formula should be applied to all subsidiaries.

## Discussion

We want to explain why the SIG became an anchor for this study. SIG is the abbreviation for Semen Indonesia Group, for which SMGR is the ticker code listed on the Indonesia Stock Exchange (BEI). The corporation is a state-owned Indonesian cement holding, the largest cement producer in Indonesia. The SIG group was an umbrella of 36 business entities and became the dominant player in Indonesia’s cement market. In 2021, SIG obtained a remarkable market share of 61.12%, with total cement sales reaching 40.47 million tons. There is still idle capacity, as the total installed capacity should be 53.55 million tons. The holding acquired Holcim Indonesia (SMCB) at the end of 2018, consolidating SIG’s position as a gigantic corporation in the region.

Because SIG is the largest cement maker, the total number of employees is ~9359 people, the largest in Indonesia, who are stationed throughout the entire nation. Some are located at headquarters in Jakarta, the capital city of Indonesia, and a more significant number of others are located in hundreds of locations in the country. The SIG applies several operational management strategies. The first is central-driven top-down management, and the second is autonomous local operating company (opco) management. Central-driven management operates by determining marketing policies, production, procurement, and labour, while local policy management handles local CSR, outsources manpower, and limits local procurements.

Due to its role as the predominant corporation, especially a well-known state-owned firm, almost all SIG policies are acknowledged to be the benchmark in all aspects of the business climate in Indonesia, especially in the cement sector. The welfare standards applied to workers in SIG have been imitated in many other cement industries in Indonesia.

**Fig. 2:**
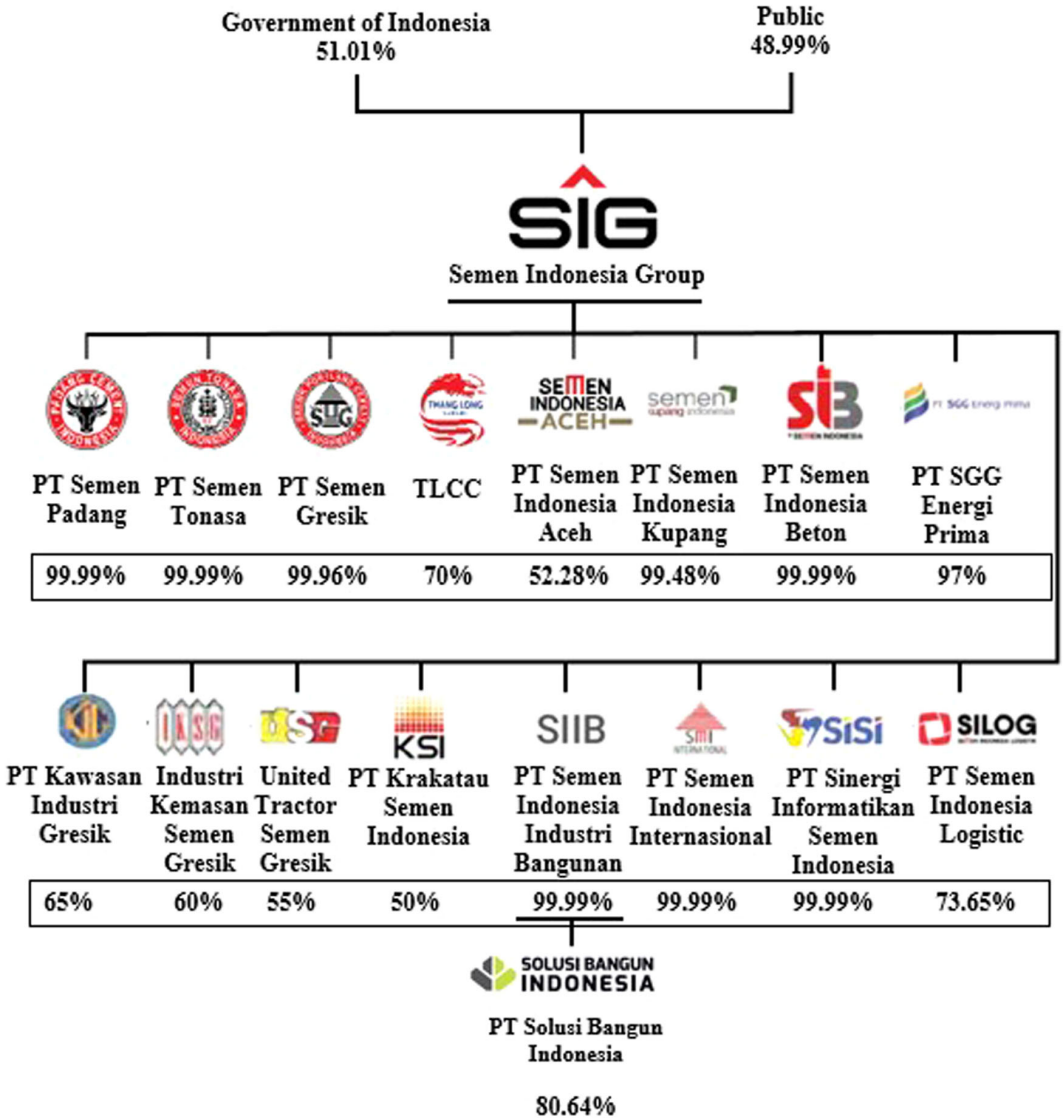
The corporate structure of SIG (2022). The SIG has total of 36 subsidiaries which is 16 companies as children as they are at second tier. Besides, SIG has 19 groups of companies at the third tier should be called business grandsons which out of scope of this study. We displayed the Solusi Bangun Indonesia (SBI) that should be one of third tier as a distinct exception, because SBI is a cement maker with a significant major market share 15% in Indonesia. The SBI is a child of the SIIB so a grandson for the SIG. Therefore, we have organized total 36’s members of SIG.

As mentioned above, Fig. 2 presents a general figure of the SIG holding. The total members of the SIG group were formed from 36 subsidiaries, but we pointed out only 17 subsidiaries, as mentioned in the figure. We did not construct the remaining 19 other subsidiaries due to size considerations. The subsidiaries are the third tier of the SIG or business grandsons, and we limited this study to only discussing the second tier or business children. The SIG itself is the parent business at the top of the business structure. We focused only on designing bonus formulations in the headquarters and subsidiaries, meaning the business grandsons were outside the scope of this study.

### Indonesian cement sectors

There are 27 groups of cement makers with 20 cement brands in Indonesia’s market in 2022. The total installed capacity reached 131.75 million tons of cement annually, while consumption gradually decreased and reached far below the numbers. This condition caused the country to experience an oversupply of cement products due to overconfidence in previous planning (Subiyanto, 2020a). The outbreak of COVID-19 first emerged at the end of 2019 and has aggravated the bitterest environment of the cement sector in Indonesia. The unbalanced figure of the cement market, marked by an increased number of cement makers while decreasing demand and unsupported by the challenging environment due to the severe pandemic, made the cement market perfect competition.

Amid this perfect competition, leading indicators such as EAT and revenue are essential to building bonus formulations. The two indicators are suitable for evaluating performance at the headquarters, as it does not have a production channel. The headquarters only report consolidation from subsidiaries’ results. The two other variables of total cement produced and operating expenses, which are operational considerations, should be relevant to analysing subsidiaries’ or opcos’ performance.

Based on Table 1, we determined clusterisation for the 27 existing corporations to introduce the welfare level given to their workers. We divided them into five clusters as peers or benchmarks using the following definitions:

1. Cluster-1 if corporations are proven to provide the highest level of welfare to their workers compared to their peers;
2. Cluster-2 if the corporations give a moderate level of welfare to their workers;
3. Cluster-3 if the corporations, on average, give welfare at a level higher than what is legally stipulated;
4. Cluster 4 if the corporations provide welfare levels as high as meeting the stipulated regulations;
5. Cluster-5 is if corporations give welfare when workers launch protests or disputes.

**Fig. 3:**
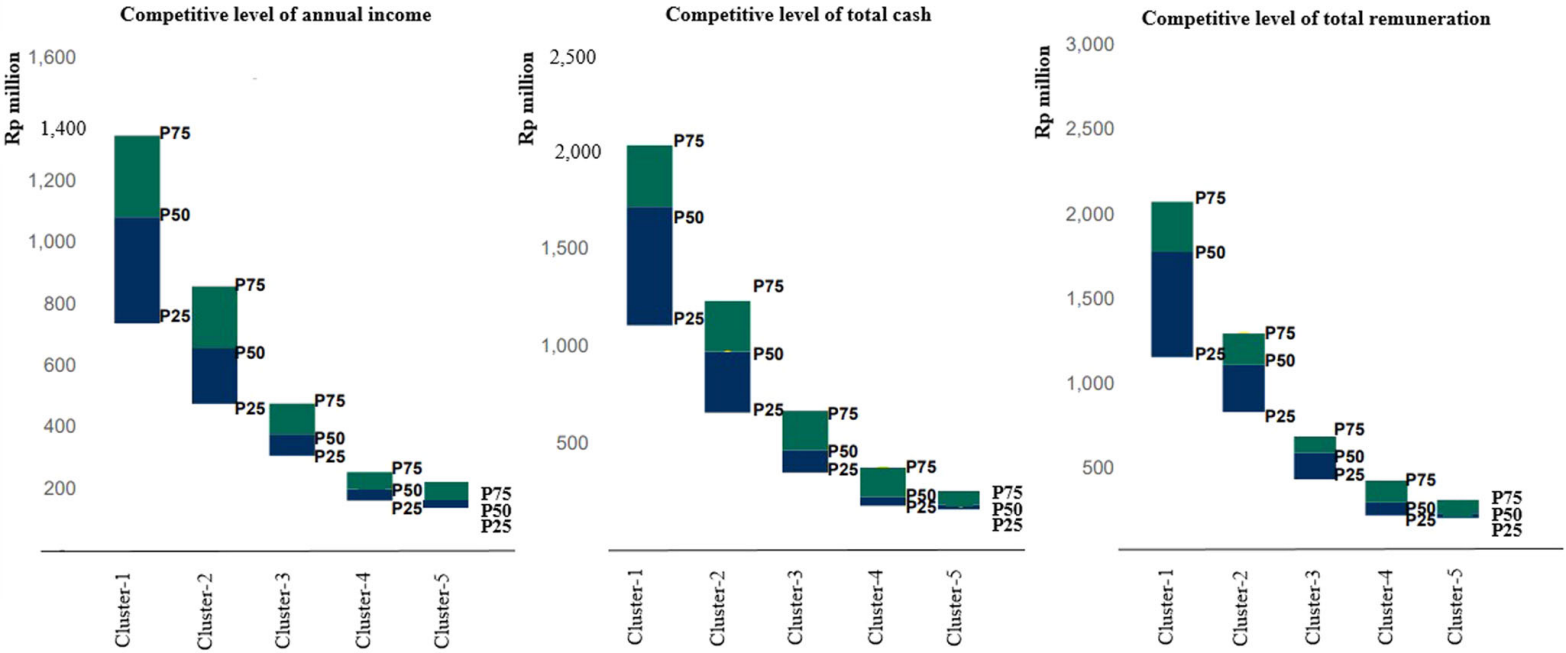
The competitive level between clusters. We distributed in three different views of competitive level of annual income, total cash, and total remuneration. Annual income is basically a normative as stipulated by each of organization, meanwhile, total cash to describe additional cash to add annual income and the sum of it is total remuneration of workers obtained.

Based on the three consecutive variables obtained from the annual reports, annual income, total cash, and total remuneration, we constructed Fig. 3. This figure shows the welfare comparison between clusters, which found that workers in cement corporations grouped in cluster-1 received much higher compensation in all aspects. The workers in the mentioned cluster were given higher pay levels than those in other clusters. This meant that workers of the cement corporations in the groups lower than cluster-1 were generally given a pay level below those in cluster-1.

The different treatments are due to the different performances. The cement corporations in cluster-1 generally matured and developed businesses in terms of their brands, markets, and products and have historically not had a conflict with their workers. Their operations were relatively stable, as were their sales.

Figure 3 shows that cluster-1 is the highest. Consecutively, the gradual level below cluster-1 is cluster-2, cluster-3, cluster-4, and cluster 5. In this case, the definition of lower is below installed capacities, lower market share, lower level of production, and a lower level of welfare given to workers. The lowest level of cluster-5 is generally the new entrants still introducing their products in Indonesia’s markets. The cluster focuses on sustaining the businesses amid stiff competition and does not hesitate to ignore workers’ rights or go against them, even when stipulated by the law.

### SIG’s bonus pool allocation

**Fig. 4:**
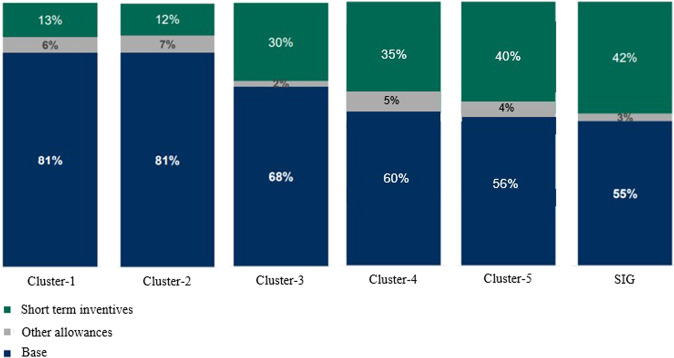
Pay mix SIG and other clusters. We construct the proportions of total remuneration for each of total 27 corporations to show that the base income of workers of bigger and better corporations is higher than other lower clusters. The companies that still developing were set high proportions of short-term incentives to compensate which are definitely not mandatory. We display profile of SIG due to the SIG is usually as an important benchmark in Indonesia.

The SIG has become the predominant classification in terms of performance indicators. The total volume of cement produced annually is the highest in Indonesia. The consolidation market share is approximately 50–60%, implying maturity in the sector yearly. However, in terms of peer categorisation, SIG is at cluster-2, according to Table 1. Generally, cluster-1 is occupied by corporations affiliated with international companies. The higher standards common in global companies have influenced local remuneration systems and Indonesia’s corporations affiliated with them, which have greatly benefited workers in terms of welfare indicators. Holcim Indonesia, for example, as part of cluster-1, was influenced by the systems developed in France. Indocement was also induced by advanced remuneration developed in Germany. Unsurprisingly, the pay level of cluster-1 was far above the average amount received by other domestic clusters. However, the pay level implemented in SIG also benefits the office, as the pay mix will reduce the burden on corporations associated with paying mandatory dues and taxes. Figure 4 shows the structure of the SIG pay mix with peers.

According to Fig. 4, the distinctive pay mix SIG unexpectedly provides many benefits for sustaining the corporation. With an identical amount of income to workers, the base income is much lower than that of peers. The SIG base income is only 55%, whereas the others were approximately 68–81%. As we are concerned, costs associated with mandatory dues and taxes are calculated based on base income stipulated by laws. The smaller the base of income, the smaller the payable amount.

In contrast, the SIG is seen as maximising nonbase incentives and allowances. Incentives and allowances, among others, are part of the annual bonuses. This meant that the SIG maintained its obligation to deliver welfare to workers at the maximum point while keeping the costs as efficient as possible.

**Fig. 5:**
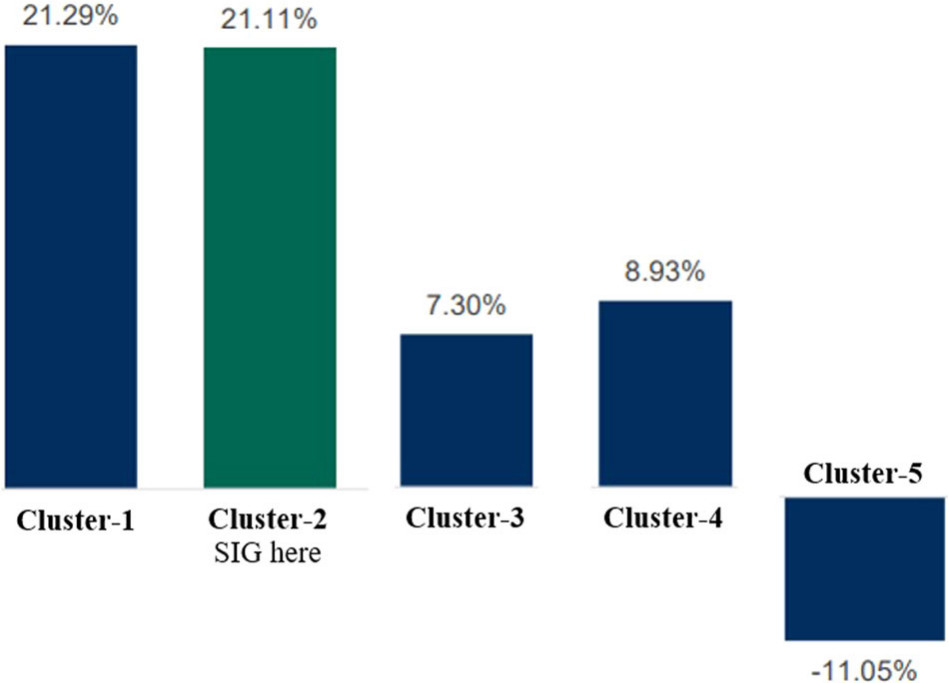
Pool allocation of annual bonuses to EAT (2021). These findings are based on exercise and evaluation and the higher clusters evidently distributed the higher bonus pool that allocated for workers.

However, the SIG has traditionally committed to meeting welfare levels above the average amount received by other local peers. The primary consideration is maintaining a better spirit among workers to be more productive and innovative and creating an excellent professional environment (Lertxundi et al., 2019; Brück et al., 2021). Based on the 2021 annual report, SIG, on average, distributed 21.11% of EAT as a bonus allocation to workers. Figure 5 shows the pool allocation between clusters and SIG. Utilising the same approaches analysed in their annual reports, we found that cluster-1 allocated 21.29% of their EAT to workers, followed by 7.3%, 8.93%, and −11.05% for cluster-3, cluster-4 and cluster-5, respectively.

**Fig. 6:**
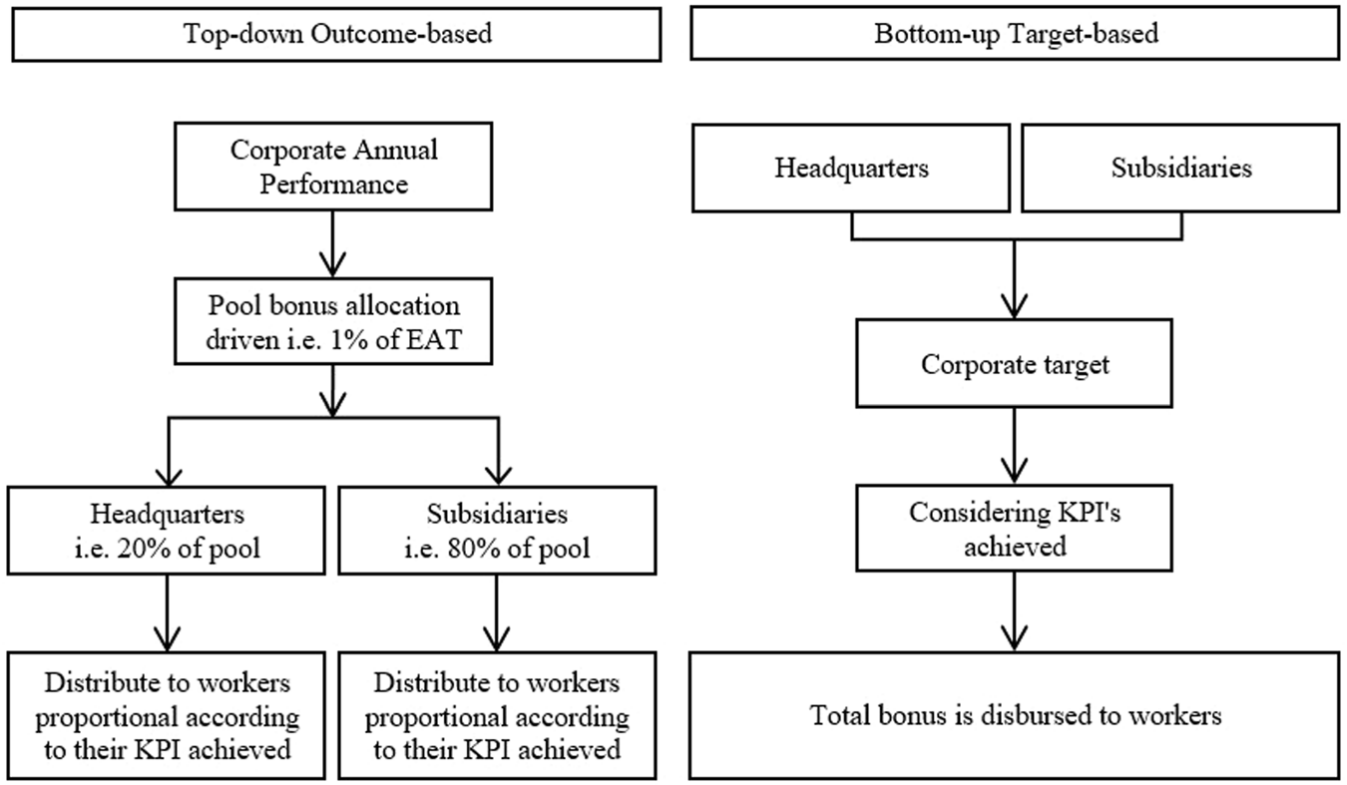
The two types of SIG strategies. The two different strategies to be carefully considered. The SIG has enacted both of techniques and the best piece of application is the bottom-up target-based.

The pooling technique employed by the SIG to achieve 21.11%, as shown in Fig. 5, resulted from implementing a bottom-up target-based strategy rather than a top-down outcome-based strategy. These two strategies are illustrated in Fig. 6. Based on practical industrial experience, the SIG regularly chooses the bottom-up target-based approach because of several considerations, as shown in Table 4.

**Table 4:** Differences in considerations to determine annual bonus pool allocation.

No.	P/C	Top-down outcome-based	Bottom-up target-based
1	Pros	Require alignment to the corporate financial performance	Easier to be managed
2		Pool allocations will be disbursed based on contributions	Higher control due to derivative from the percentage of EAT
3			Key performance index (KPI) driven
4			More transparency
5	Cons	Higher volatile the amount of pool	Lowering the opportunity to optimise fixed cost
6		Require to reconcile between HR and finance	Require strong alignment to KPI
7		Unilateral	

The top-down outcome-based mentioned on the left side of Fig. 6 implies a command-driven by the headquarters’ office to control the groups firmly. This style is unilateral, as headquarters highly determine the amount pool without any initial information and suitable formulation. This type of strategy frequently triggers disputes and conflicts among workers and unions versus management because the measure of the amount was not fair enough to appreciate their efforts and sacrifices (Aqqad et al., 2019). It can be assured that workers and unions will demand appropriate formulations. The questions sparked are how much of the pool was allocated and how it was developed.

The right side of Fig. 6 accommodates the voices of workers and unions from the bottom line. The treatment is much fairer, as the bottom-up target-based approach comes from the results achieved and then upscaled as a calculation basis to determine a decent annual pool bonus. Thus, this technique has successfully silenced disputes and conflicts; consequently, the industrial climate and environment are more stable in maintaining productivity.

### Determining the percentile system

**Fig. 7:**
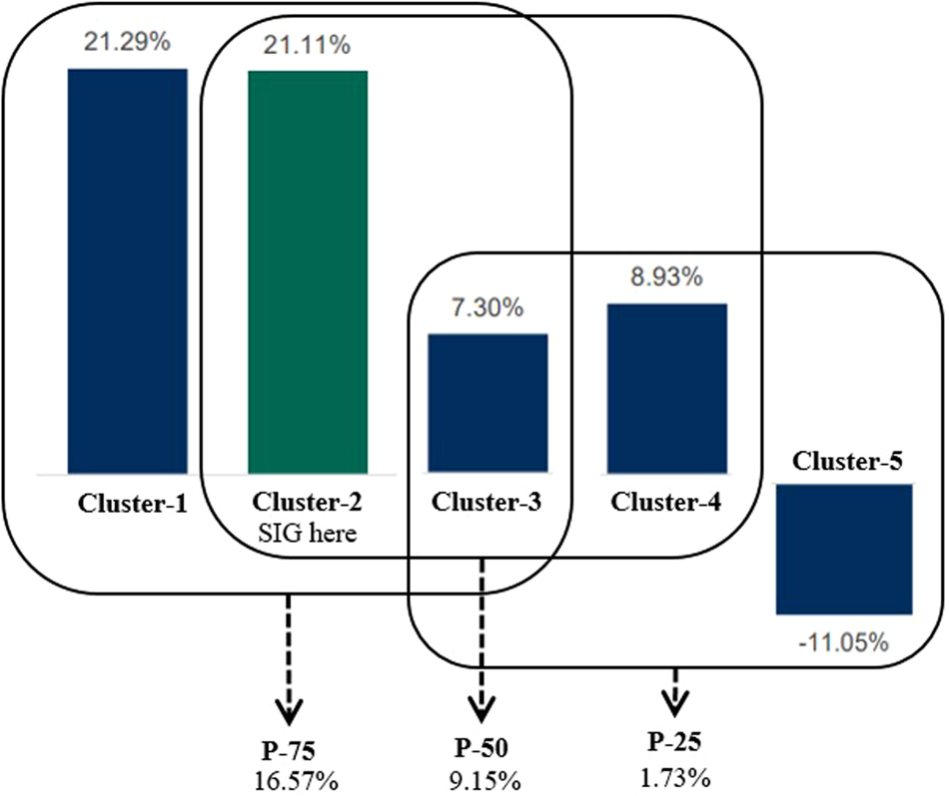
Determining percentile methods of P-25, P-50, and P-75. To resolve industrial conflicts, we determine three percentile methods to distribute bonuses. The corporate must apply P-25 that is evidently still in developing phase, but the higher or better corporations must follow the higher percentile of P-50 or P-75.

Percentile (P) methods were obtained from the analyses of annual reports. According to Table 3, the cement makers have been grouped into each cluster, implying the total remunerations given to their workers. Figure 5 shows the average pool allocation for each cluster. Based on these figures, this study suggests applying percentile methods as empirical cases divided into three systems denoted as P-25, P-50, and P-75. Each *P* resulted from considerations of the scale of the total remuneration received. This study produces a percentile method in Table 5 as the results obtained by assessing the total remuneration for workers from all of Indonesia’s cement corporations. P-25 resulted from the average of the three bottom peers, whereas P-75 resulted from the top three peers. P-50, the middle percentile, resulted from three peers in the middle of the five peers surveyed. Figure 7 shows the technique used to determine the percentile methods.

**Table 5:** SIG’s simulation to obtain annual bonus pool allocation in 2021.

Description	P-25	P-50	P-75	Average
Percentage of EAT	1.73	9.15	16.57	9.52
SIG’s annual bonus pool (US$ million)	Not-apply	12.79	Not-apply	Not-apply

Based on Fig. 7, we can simulate the bonus pool allocation for the real role. Considering SIG’s total earnings after taxes (EAT) 2021 reached Rp 2,014 billion (US$ 139.86 million), there will be three options as a choice and alternative. The first was the utilisation of P-25, P-50, or P-75. The workers represented by the union can offer the best suit because SIG is classified as cluster-2; therefore, the option should be P-50. The SIG’s workers, in this case, must receive an annual bonus pool allocation of 9.15% of the EAT, or the amount will be US$ 12.79 million for 2021. Table 5 explains the result.

However, the final value obtained depends on the views and considerations of BOCs or BODs. It also depends on the bargaining position of the workers and the union. According to peers, the SIG is part of cluster-2; however, the final agreement between management and workers or unions might choose one step higher or even remain on the line. Certain corporations may choose a wiser option by deciding an average of 9.52%, according to the calculation made in Table 5, which looks like a win-win solution. This case depends on discussion and communication among workers, unions, and management (McCann and Allen, 2021). The chosen percentage must be respected as a mutual decision between the parties involved.

### Developing bonus formulation

After the annual bonus pool has been determined, further calculations measure the adequacy of the bonus allocation for headquarters and subsidiaries. The detailed weighing of business sectors must be mutually agreed upon from the beginning of the year. For example, SIG is divided into two sectors: cement and noncement makers. For the cement sector, as the primary business, the portion allocation could be stated as 80%, and the remaining 20% for the noncement sectors. These percentages are taken based on the size of the given contribution as the total revenue they delivered. Based on annual reports, cement makers contributed 80% of total revenue, while noncement makers provided the rest. The decision depends on specific considerations and is distinct but should be determined as mutually agreed upon at the beginning of the year. Unfortunately, this study does not discuss this issue; therefore, the authors propose to extend the scope of future research.

As previously stated, headquarters and subsidiaries have different climates. The headquarters do not have the machinery for operation and production, but this office has intangible strategies and policies. In this case, the EAT consolidation is appropriate for being considered one of the variables to develop the bonus formulation at headquarters. In addition, this central office communicates with investors and business relations; therefore, EAT is adequately claimed for headquarters. Another variable to be considered in the headquarters’ portion is revenue. Table 6 shows that the KPI score for headquarters was 104.9% after two indicators were enacted.

**Table 6:** Annual bonus formulation for headquarters.

No.	Variables	UoM	Scale of weight (%)	Planning	Realisation	Achievement (%)	Final score (%)
			A	B	C	D	E = D × A
1	EAT	US$ million	60	21.69	23.31	107	64.5
2	Revenue	US$ million	40	362.87	367.09	101	40.5
			100	KPI Score			104.9

According to Table 6, we build a bonus formulation for headquarters as follows: 0.6EAT + 0.4 Revenue. Then, we have to determine the weight scale; in this case, 60% and 40% proportions are acceptable based on the annual report assessment and mutual agreement. In addition, if the deepest concern is net profit, it will be adequate if EAT sets a higher weight than revenue. Subsequently, we can measure achievement calculated based on realisation/planning, and the final score is the final score of KPI.

Using the same methods, we included two variables, COGM and operational expenses, to calculate the KPI score for subsidiaries or opcos. Table 7 shows the subsidiaries’ KPI scores; the final score was 108.8%.

**Table 7:** Annual bonus formulation for subsidiaries.

No.	Variables	UoM	Scale of weight (%)	Planning	Realisation	Achievement (%)	Final score (%)
			A	B	C	D	E = D × A
1	COGM	US$ million	60	351,185	346,360	101	61
2	Operational expenses	US$ million	40	129,506	90,843	120	48
			100	KPI Score			108.8

According to Table 7, the bonus formulation for subsidiaries is 0.6 COGM + 0.4 Operational expenses. Performance is calculated based on realisation/planning, and the result is the final score of the KPI. Determining the weight of 60% and 40% must be a mutual agreement between parties. If the deepest concern is cost, it will be adequate if COGM sets a higher weight than operational expenses. This table concerns the costs; therefore, the higher the performance, the lower the costs and vice versa.

Furthermore, we utilise the values from Tables 6 and 7 in Table 8 to determine the number of portions achieved for each entity between the headquarters and subsidiaries. As mentioned, we simulate the headquarters weight factor for 20% and 80% for the subsidiaries. The final achievement for headquarters was 104.9%*20%/100 = 21%, while that for subsidiaries was 108.8%*80%/100 = 87%. The result in Table 8 may be over 100%; in this case, we propose letting the number open a chance for an additional negotiating bonus because of the extraordinary efforts made. This consideration was intended to build better industrial relationships.

**Table 8:** Determining the portion between headquarters and subsidiaries.

Description	SIG-Headquarter	Subsidiaries
KPI Score (%)	104.9	108.8
Weight factor (%)	20	80
Final achievement (%)	21	87

Finally, we build a better model based on adequate knowledge to develop a fair annual bonus formulation. Based on the percentile results, this study has decided to place a yearly bonus allocation of US$ 12.79 million in 2021. Table 9 shows that the annual bonus in 2021 for headquarters should be US$ 2.51 million; on the other hand, the yearly bonus for subsidiaries will be US$ 10.28 million. This result was calculated based on the headquarters weight factor of 20% and the subsidiaries or opcos weight factor of 80%.

**Table 9:** Final annual bonus allocation between headquarters and subsidiaries.

Description	SIG-headquarter	Subsidiaries
Annual bonus pool obtained (US$ million)	2.51	10.28

Last, because the total number of SIG’s subsidiaries is 36, the amount of US$ 10.28 million must be equally distributed as a fair bonus payment for all subsidiaries. The methods are certainly applied based on the performance results they achieved.

## Conclusion

The SIG, the largest cement producer in Indonesia, is a prestigious state-owned cement because of its achievements. Although almost all indicators have become benchmarks for other cement producers in Indonesia, the SIG’s workers’ total remuneration is classified as part of cluster-2 instead of cluster-1. This means that the total welfare given to workers would be approximately slightly below that received by workers in cluster-1. Interestingly, although the pay mix of SIG is quite different from that of equal peers, the other peers in this context emulate the SIG annual bonuses formulation to be applied in their offices.

SIG, on the other hand, based on peers’ considerations, urgently needs to calibrate its package remuneration to be identical to other clusters. A base income of approximately 55% compared to others is insufficient to assure welfare when workers retire. Since state-owned firms have been proven to contribute significantly to the nation, an increased income basis is urgently needed.

Each corporation should consider recent achievements to determine the best percentile method for annual bonus pooling. This achievement will put corporations in the right place of the percentile as a subject matter to be discussed between parties, the workers and management. The greater the percentage of EAT given as annual bonuses, the greater the chances for workers to improve their welfare. Management must acknowledge that a more significant portion of welfare allocated also fuels the spirit of workers to improve their productivity and, ultimately, corporate performance.

Practically, percentile systems have been proven and implemented in SIG. The percentiles were divided into three groups: P-25, P-50, and P-75. Other cement corporations may imitate these methods, but the final decision will be subject to mutual agreement between workers and management. This percentile decision is imperative for determining the annual bonus pool allocation to meet workers’ rights.

The ultimate goal is to develop a fair annual bonus formulation that should be applied at headquarters and subsidiaries. These findings proposed EAT and revenue as variables to calculate the headquarters’ KPI based on empirical data. In contrast, the two other variables of COGM and operating expenses should measure the subsidiaries’ or opcos’ KPI. These KPIs are, in this study, limited to a headquarters’ KPI and subsidiaries’ KPI, while the KPI of each worker shall be detailed in different measurements. This detailed subject is an open issue for future research.

## Data Availability

Data are included in the body of this study.
